# Glycolysis is essential for chemoresistance induced by transient receptor potential channel C5 in colorectal cancer

**DOI:** 10.1186/s12885-018-4123-1

**Published:** 2018-02-20

**Authors:** Teng Wang, Kuan Ning, Xu Sun, Chun Zhang, Lin-fang Jin, Dong Hua

**Affiliations:** 1 Department of Oncology, Affiliated Hospital of Jiangnan University, Wuxi, Jiangsu 214062 China; 2 Wuxi Medical College, Jiangnan University, Wuxi, Jiangsu 214122 China; 3 Department of Pathology, Affiliated Hospital of Jiangnan University, Wuxi, Jiangsu 214062 China

**Keywords:** Colorectal cancer, Chemoresistance, Transient receptor potential canonical C5, Glycolysis, Intracellular Ca^2+^

## Abstract

**Background:**

Elevated intracellular Ca^2+^ ([Ca^2+^]_*i*_) level could lead to [Ca^2+^]_*i*_ overload and promote apoptosis via different pathways. In our previously study, up-regulated expression of transient receptor potential canonical channel (TRPC5) was proven to increase [Ca^2+^]_*i*_ level, and resulted in chemoresistance whereas not apoptosis in human colorectal cancer (CRC) cells. The ATP-dependent homeostatic maintenance of resting [Ca^2+^]_*i*_ should be important in this process. Increased glycolysis was found to be an important adenosine triphosphate (ATP) source in cancer. This study aimed to explore the potential mechanism of aerobic glycolysis in transient receptor potential channel TRPC5 induced chemoresistance.

**Methods:**

In this study, we examined glucose transporter 1 (GLUT1) expression, glucose consumption and celluar ATP production to determine glycolytic activity. Real-time PCR and western blot were analyzed to determine TRPC5 expression at the mRNA and protein levels in human CRC cells (HCT-8, LoVo), and fluorouracil (5-Fu) resistant CRC cells (HCT-8/5-Fu, LoVo/5-Fu). 3-bromopyruvate (3-BP) and 2-Deoxy-D-glucose (2DG) were used to inhibit glycolysis. Glycolytic activity, intracellular Ca^2+^ ([Ca^2+^]_*i*_) and the half maximal inhibitory concentration of 5-Fu (5-Fu IC50) were measured. Western blot was analyzed to determine cleaved Caspase-3 protein level. Flow cytometry was performed to detect the apoptosis rates. Immunohistochemistry staining was performed to determine TRPC5 and GLUT1 expression level in human CRC tissues.

**Results:**

Overproduced of TRPC5 and increased glycolysis were found in HCT-8/5-Fu and LoVo/5-Fu than in HCT-8 and LoVo cells. Compared to HCT-8 cells, the HCT-8/5-Fu cells showed higher [Ca^2+^]_*i*_ levels which decreased after treated with TRPC5-specific shRNA. Furthemore, inhibition of glycolysis resulted in decreased ATP production, elevation of [Ca^2+^]_*i*_ level and cleaved caspase-3, increased apoptotic cells rate, and a remarkable reversal of 5-Fu resistance in HCT-8/5-Fu cells, while showed no effect in HCT-8 cells. BAPTA-AM, a [Ca^2+^]_*i*_ chelator, could reduce the elevation of cleaved caspase-3 and increased apoptotic cells rate due to glycolysis inhibition. Advanced CRC patients with high expression of TRPC5/GLUT1 displayed poorer chemotherapy outcome, and notably, the significant association between high TRPC5 expression and chemoresistance is GLUT1 expression level dependent.

**Conclusions:**

We demonstrated the essential role of glycolysis in TRPC5 induced chemoresistance in human CRC cells via maintaining [Ca^2+^]_*i*_ homeostasis.

## Background

Colorectal cancer (CRC) is one of the most common malignant tumors and showed a high cancer-related death in China [1]. Chemotherapy is the main treatment for CRC patients. Resistance to chemotherapy occurs in most cases, which results in treatment failure. Intracellular Ca^2+^ ([Ca^2+^]_*i*_) is reported to be involved in diverse cellular biological behaviors. Transient receptor potential canonical channel 5 (TRPC5) is a Ca^2+^**-**permeable channel that could modulate [Ca^2+^]_*i*_ level. In our previously study [2], TRPC5 was proven to activate Wnt/β-catenin signal pathway and induce chemoresistance. The [Ca^2+^]_*i*_ that could be increased by TRPC5, acts as “double-edged sword” in cellular process. At different levels, it not only participates in cell proliferation, differentiation and gene transcription, but also induces cell apoptosis [3]. Hence, the maintenance of [Ca^2+^]_*i*_ homeostasis might be important in TRPC5 induced chemoresistance.

[Ca^2+^]_*i*_ efflux is an energy dependent activity [4–6]. Altered energy metabolism in malignant tumor is one of the hallmarks of malignancies [7]. Actually, even in the presence of ample oxygen, cancer cells prefer to metabolize glucose by glycolysis (aerobic glycolysis) [8]. Several studies showed aerobic glycolysis was an important source of adenosine triphosphate (ATP) production in cancer cells [4, 9, 10], and glycolytic ATP is of great importance for [Ca^2+^]_*i*_ efflux and in maintaining a low resting [Ca^2+^]_*i*_ [4, 11]. Here, we designed a study to explore the potential mechanism of aerobic glycolysis in TRPC5 induced chemoresistance.

## Methods

### Cells and cell culture

The wild human CRC cell line HCT-8 (KG028) and LoVo (SCSP-514) were purchased from Keygen Biotech Co. Ltd. (Nanjing, Jiangsu Province, China) and the Cell Resource Center of Shanghai Institutes for Biological Sciences, Type Culture Collection of the Chinese Academy of Sciences (Shanghai, China) respectively. Fluorouracil (5-Fu)-resistant HCT-8 cells (HCT-8/5-Fu) (KG333) was purchased from Keygen Biotech Co. Ltd. 5-Fu-resistant LoVo cells (LoVo/5-Fu) were derived by treating LoVo cells with stepwise increasing concentrations of 5-Fu (Jinyao Amino Acid Co. Ltd., Tianjin, China) over 6 months. The wild human CRC cells and 5-Fu-resistant CRC cells were cultured as we reported previously [2].

### Cell transfection

HCT-8/5-Fu cells on 50–70% confluence were treated with TRPC5-shRNA (sc-42,670, Santa Cruz Biotechnology, Dallas, TX, USA) (HCT-8/5-Fu/RNAi) (scrambled siRNA as control, HCT-8/5-Fu/Scrambled). 3-bromopyruvate (3-BP) (SML2000, Sigma Chemical Co., St. Louis, MO, USA) (40 μM, 24 h) or 2-Deoxy-D-glucose (2DG) (D8375, Sigma Chemical Co.) (20 mM, 24 h) was used to inhibit the glycolysis. If needed, cells were treated with BAPTA-AM (A1076, Sigma Chemical Co.) (20 μM, 1 h) before glycolysis inhibition. Expression of TRPC5 and GLUT1 were deternmined by Real-time PCR and western blot.

### Western blot

Whole-cell protein was obtained using RIPA containing 1 mM PMSF. An equal quantity of total proteins was electrophoresed on 8% polyacrylamide gel containing 0.1% SDS and then transferred to PVDF membrane. After blocked with phosphate-buffered saline tween containing 5% non-fat milk, the PVDF membranes were incubated with the primary antibodies anti-TRPC5 (ACC-020, Alomone labs, Jerusalem, State of Israel) (1:500), anti-caspase-3 (ab32351, Abcam Biotechnology, Cambridge, MA, USA) (1:500), anti-glucose transporter 1 (GLUT1) (ab115730, Abcam Biotechnology) (1:1000), β-actin (AA128, Beyotime Biotechnology) (1:1000) and subsequently with the corresponding secondary antibodies [goat anti-rabbit IgG (A0208, Beyotime Biotechnology) and goat anti-mouse IgG (A0216, Beyotime Biotechnology)]. The bands were quantified using ImageJ software (NIH, Bethesda, MD). β-actin was used as the internal control for normalization.

### Real-time PCR

TRIzol (10296–010, Camarillo, CA, USA) was used to extract total RNA from cells. Real-time PCR and the comparision of the mRNA levels were performed according to the reported study [2]. Table 1 listed the primer pairs used in this study.

**Table 1:** Real-time PCR primers

Gene	Forward primer sequence (5′-3′)	Reverse primer sequence (5′-3′)
TRPC5	CCACCAGCTATCAGATAAGG	CGAAACAAGCCACTTATACC
GLUT1	CTTTGTGGCCTTCTTTGAAGT	CCACACAGTTGCTCCACAT
β-actin	GCCCTTGCTCCTTCCACTATC	CCGGACTCTTCGTACTCATCCT

### MTT assay

Twelve hours after 10^4^ CRC cells (200 μl) seeded in 96-well plates, the cells were treated with 5-Fu of different concentrations. After 48 h, the cells in each well were incubated with resh RMPI1640 (200 μl) containing 3-(4, 5-dimethylthiazol-2-yl)-2, 5-diphenyl tetrazolium bromide (MTT) (M2128, Sigma Chemical Co.) (5 mg/ml) for 4 h. Dimethyl sulfoxide (DMSO) (D8418, Sigma Chemical Co.) (150 μl) was added to each well and then the absorbance was detected at 490 nm.

### [Ca^2+^]_*i*_ measurement

We used GECO1.2 (a calcium indicator) to measure [Ca^2+^]_*i*_ level. The fluorescence signals of GECO1.2 reflected the [Ca^2+^]_*i*_ levels. The detailed procedure was in accordance with the previously reported study [2].

### Glucose consumption measurement

About 1 × 10^6^ cells were seeded in 6-well cell culture microplates. The medium was replaced with 3 ml RMPI-1640 without fetal calf serum the next day. Twenty four hours later, the medium was collected and the glucose concentration in the medium was determined according to Glucose (HK) kit (GAHK-20, Sigma Chemical Co.). Glucose consumption rate was defined as the ratio of the glucose concentration after twenty four hours divided by the glucose concentration before twenty four hours.

### Cellular ATP measurement

On reaching 50–70% confluence, ells seeded in 6-well cell culture microplates were treated with lysis reagent to release ATP. The supernatant was obtained to measure ATP according to the manufacturer’s protocol (S0026, Beyotime Biotechnology).

### Apoptosis measurement

Quantitation of apoptotic cells was obtained using the Annexin V-FITC/PI Apoptosis Detection Kit (C1062, Beyotime Biotechnology) according to the manufacturer’s protocol. Cells in logarithmic phase were detached to obtain a single cell suspension. After a total of 1 × 10^6^ cells were washed in PBS for 2 times, 195 μL of binding buffer solution was added for cell resuspension. Then 5 μL of annexin V-FITC and 10 μL of PI were added into culture solution for mixing, with incubation 30 min at 4 °C. Flow cytometry was used to make a comparison of the apoptotic cells ratio.

### Patients and immunohistochemistry staining

Ethical permission was obtained from the Ethics Committee at the Affiliated Hospital of Jiangnan University and conformed to the provisions of the Declaration of Helsinki (as revised in Fortaleza, Brazil, October 2013). The advanced CRC patients who received a biopsy and/or surgery for a primary lesion and postoperatively 5-Fu based first-line systematic chemotherapy at the Affiliated Hospital of Jiangnan University from January 2010 to December 2016 were enrolled in this study. The exclusion criteria was according with our previous study [12]. Treatment response was evaluated according to the Response Evaluation Criteria in Solid Tumors 1.1 (RECIST 1.1) guideline [13] after 2 cycles of chemotherapy. Patients achieved progressive disease (PD) or stable disease (SD) were considered as non-responders, and patients achieved partial response (PR) or complete response (CR) were considered as responders. Immunohistochemistry staining was performed to detect TRPC5 and GLUT1 protein expression in CRC tissue slides with the procedure we reported previously [12]. The results were judged according to German semi-quantitative scoring system [14] (no staining = 0; weak staining = 1, moderate staining = 2, strong staining = 3) and the extent of stained cells (0% = 0, 1–24% = 1, 25–49% = 2, 50–74% = 3, 75–100% = 4). The final score was determined by multiplying the intensity score with the extent score, ranging from 0 to 12. Each grade of TRPC5 and GLUT1 were from the same sample.

### Statistical analysis

The most appropriate cutoff values of TRPC5 and GLUT1 score were obtained by generating receiver operating characteristics (ROC) curve. The results are presented as mean ± standard error. Statistical significance was determined by a Student’s t-test, one-way ANOVA and a Pearson’s chi-squared test as applicable. A value of *p* < 0.05 was considered statistically significant. Statistical analysis was done using SPSS (version 20).

## Results

### Up-regulated of TRPC5 expression and increased glycolysis in 5-Fu chemoresistant human CRC cells

**Fig. 1:**
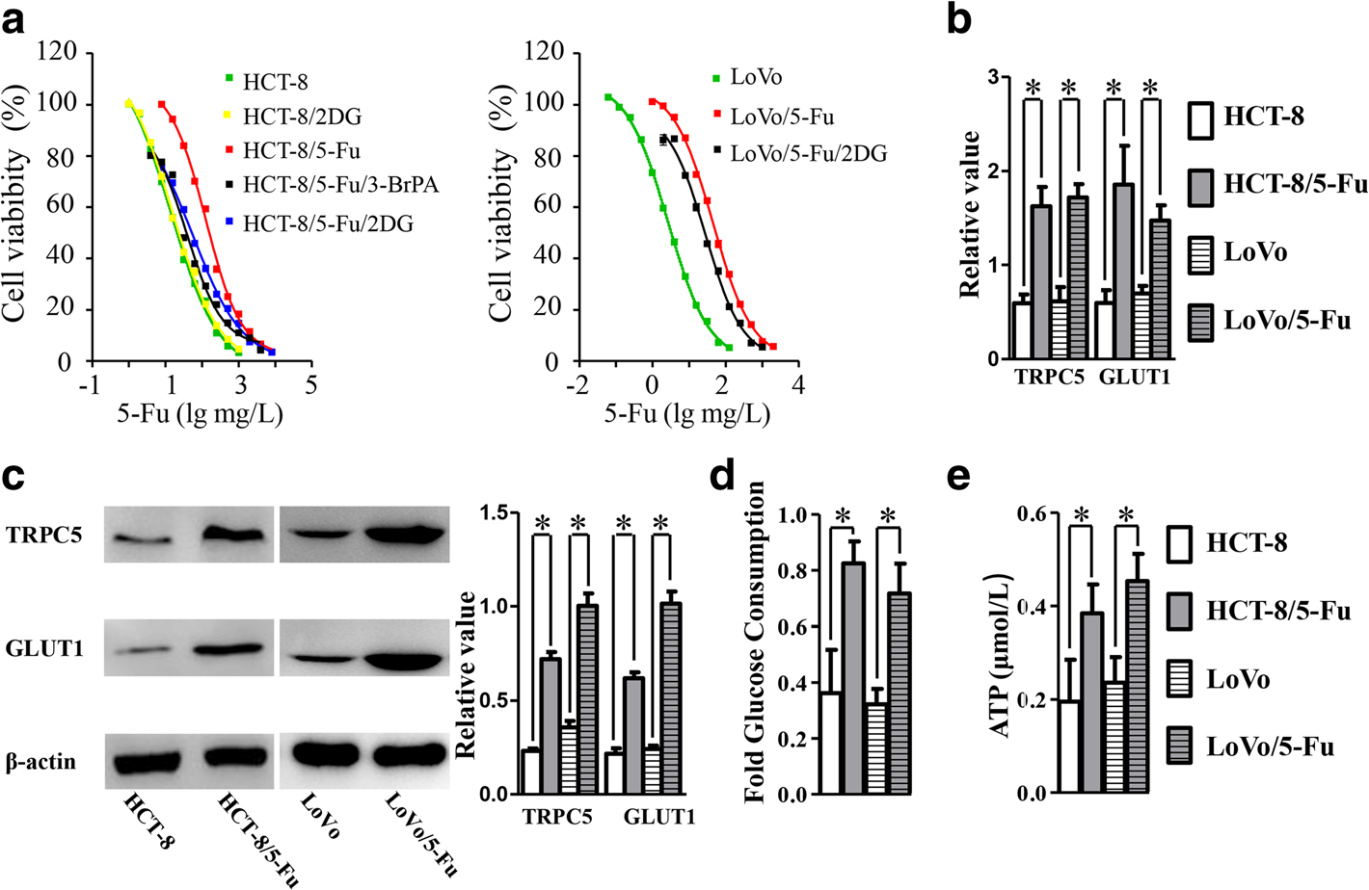
Up-regulated of TRPC5 expression and increased glycolysis in 5-Fu chemoresistant human CRC cells. **a** MTT assay showed that HCT-8/5-Fu and LoVo/5-Fu cells were much more resistant to 5-Fu-induced cell death than HCT-8 and LoVo cells. Administration of 3-BP or 2DG caused a remarkable reversal of 5-Fu resistance in HCT-8/5-Fu and LoVo/5-Fu cells, while caused no obvious change to HCT-8 cells (*n* = 6, *p < 0.05, Student’s t-test). Real-time PCR (**b**) and western blot (**c**) showed much higher expression of both TRPC5 and GLUT1 at the mRNA and protein levels in HCT-8/5-Fu than in HCT-8 cells (*n* = 4, *p < 0.05, Student’s t-test). **d** Higher glucose consumption rates in HCT-8/5-Fu cells than in HCT-8 cells (*n* = 4, *p < 0.05, Student’s t-test). **e** More ATP production in HCT-8/5-Fu than in HCT-8 cells (*n* = 4, *p < 0.05, Student’s t-test)

The MTT assay was performed to determine the half maximal inhibitory concentration of 5-Fu (5-Fu IC50) of the CRC cells. HCT-8/5-Fu (5-Fu IC50: 122.3 mg/L) and LoVo/5-Fu (5-Fu IC50: 44.76 mg/L) showed more resistance to cytotoxicity of 5-Fu than in HCT-8 (5-Fu IC50: 13.8 mg/L) and LoVo (5-Fu IC50: 2.611 mg/L) cells (Fig. 1a). Further real time PCR and western blot showed a much higher expression of TRPC5 in HCT-8/5-Fu and LoVo/5-Fu cells than their parental lines (Fig. 1b, c). Overproduced GLUT1 was reported to be essential for the increased glucose import in aerobic glycolysis in cancer [7, 15, 16]. In this study, glycolytic activity was determined by examination of GLUT1 expression, glucose consumption and celluar ATP production. Real-time PCR and western blot showed a much higher expression of GLUT1 in 5-Fu-resistant CRC cells than in their parental lines (Fig. 1b, c). Additionally, 5-Fu-resistant CRC cells showed higher glucose consumption rates and more ATP production than the wild type cells (Fig. 1d, e).

### Up-regulated TRPC5 expression induces elevated [Ca^2+^]_*i*_ level in 5-Fu chemoresistant human CRC cells

**Fig. 2:**
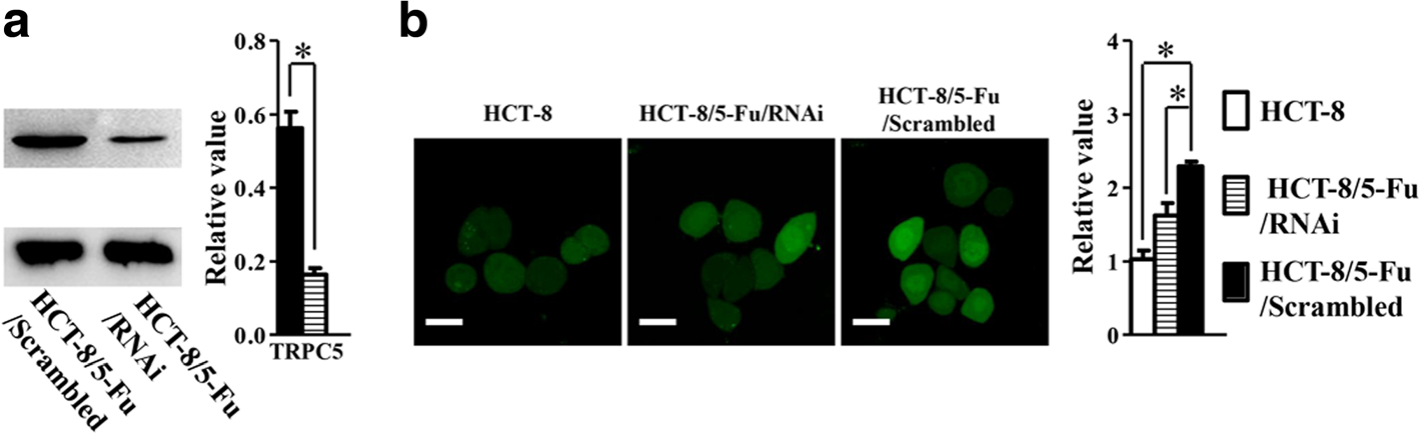
**a** Inhibition of TRPC5 by shRNA resulted in decreased TRPC5 protein expression (*n* = 4, *p < 0.05, Student’s t-test). **b** Up-regulated TRPC5 expression induces elevated [Ca^2+^]_*i*_ level in 5-Fu chemoresistant human CRC cells. The level of [Ca^2+^]_*i*_ in HCT-8/5-Fu/Scrambled cells was higher than in HCT-8 cells and dramatically decreased after the inhibition of TRPC5 by shRNA. (*n* = 4, *p < 0.05, one-way ANOVA) (Scrambled: scrambled shRNA). Scale bars, 20 μm

The roles of trp channels in cancer include changes in [Ca^2+^]_*i*_ level [17]. TRPC5 is a nonselective cation channel with Ca^2+^ permeability [18]. In our previous study [2], TRPC5 was proven to be required for the increase of [Ca^2+^]_*i*_ in HCT-8/5-Fu cells. In present study, according with the up-regulated expression of TRPC5, the level of [Ca^2+^]_*i*_ in HCT-8/5-Fu cells was higher than in HCT-8 cells. Further inhibition of TRPC5 by shRNA resulted in decreased TRPC5 protein expression (Fig. 2a) along with dramatically decreased [Ca^2+^]_*i*_ level (Fig. 2b).

### Glycolysis is crucial for preventing [Ca^2+^]_*i*_ overload in chemoresistance induction by TRPC5

**Fig. 3:**
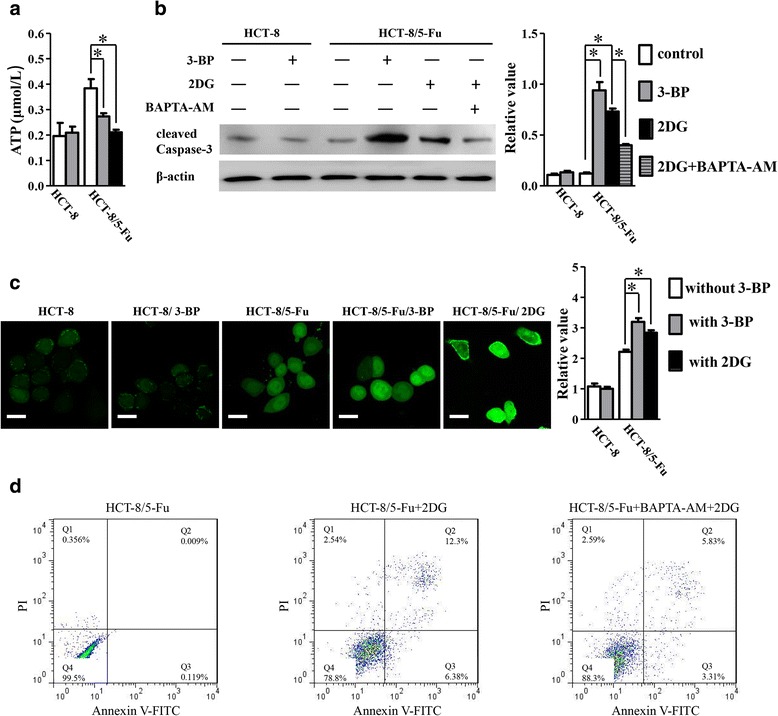
Administration of 3-BP or 2DG caused a remarkable ATP production decrease (**a**) and cleaved Caspase-3 increasement (**b**) in HCT-8/5-Fu cells, while caused no obvious change to HCT-8 cells (**a**, **b**) (*n* = 4, *p < 0.05, one-way ANOVA). The elevated cleaved Caspase-3 induced by 2DG decreased dramatically with the administration of BAPTA-AM (**b**). (*n* = 4, *p < 0.05, one-way ANOVA). (**c**) Administration of 3-BP or 2DG caused increasement of [Ca^2+^]_*i*_ level in HCT-8/5-Fu cells, while caused no obvious change to HCT-8 cells (*n* = 4, *p < 0.05, one-way ANOVA). Scale bars, 20 μm. **d** The apoptotic cells rate in HCT-8/5-Fu cells increased dramatically after treated with 2DG. The elevated apoptotic cells rate induced by 2DG decreased dramatically with the administration of BAPTA-AM (*n* = 4)

Several studies showed up-regulated expression of trp proteins [3] could lead to [Ca^2+^]_*i*_ overload, which was demonstrated to induce apoptosis [3, 19, 20]. With regard to the augmentation of Ca^2+^ influx through up-regulated expression of TRPC5, ATP-dependent Ca^2+^ efflux should be crucail to prevent [Ca^2+^]_*i*_ overload related apoptosis. Since reprogramed energy metabolism to glycolysis was demonstrated to be the major mechanism of generating ATP [4, 7, 21] and the major ATP source for Ca^2+^ efflux in cancer [4], we explored the potential mechanism of [Ca^2+^]_*i*_ homeostasis in chemoresistance induction by TRPC5. 3-BP and 2DG, inhibitors of glycolysis [10, 22], were used to inhibit glycolysis in human CRC cells. Administration of 3-BPor 2DG caused a remarkable ATP production decrease and increasement of [Ca^2+^]_*i*_ level in HCT-8/5-Fu cells, while caused no obvious change in ATP production and [Ca^2+^]_*i*_ level in HCT-8 cells (Fig. 3a, c). In addition, western blot showed administration of 3-BP or 2DG increased cleaved Caspase-3 in HCT-8/5-Fu cells significant, while showed little change of cleaved Caspase-3 level in HCT-8 cells (Fig. 3b). FCM showed administration of 2DG apoptotic cells rate obviously increased in HCT-8/5-Fu cells (Fig. 3d). Further study showed the elevated cleaved Caspase-3 and apoptotic cells rate induced by 2DG dramatically decreased with the administration of BAPTA-AM (Fig. 3b, d). MTT assay showed the 5-Fu IC50 of HCT-8/5-Fu cells treated with 3-BP (HCT-8/5-Fu/3-BP) or 2DG (HCT-8/5-Fu/2DG) decreased to 44.7 ng/ml (95%CI: 36.8–48.6 mg/L) and 38.48 ng/ml (95%CI: 30.64 to 48.33 mg/L), while no significant change of 5-Fu IC50 of HCT-8 cells treated with 3-BP (HCT-8/3-BP) was observed (Fig. 1a). Similar results were obtained in experiments in LoVo/5-Fu cells. The 5-Fu IC50 of LoVo/5-Fu cells dramatically decreased to 27.77 mg/L (95%CI: 23.92–32.23 mg/L) in LoVo/5-Fu/2DG cells (Fig. 1a).

### The association of high TRPC5 expression with chemoresistance was GLUT1 expression dependent in advanced CRC

**Fig. 4:**
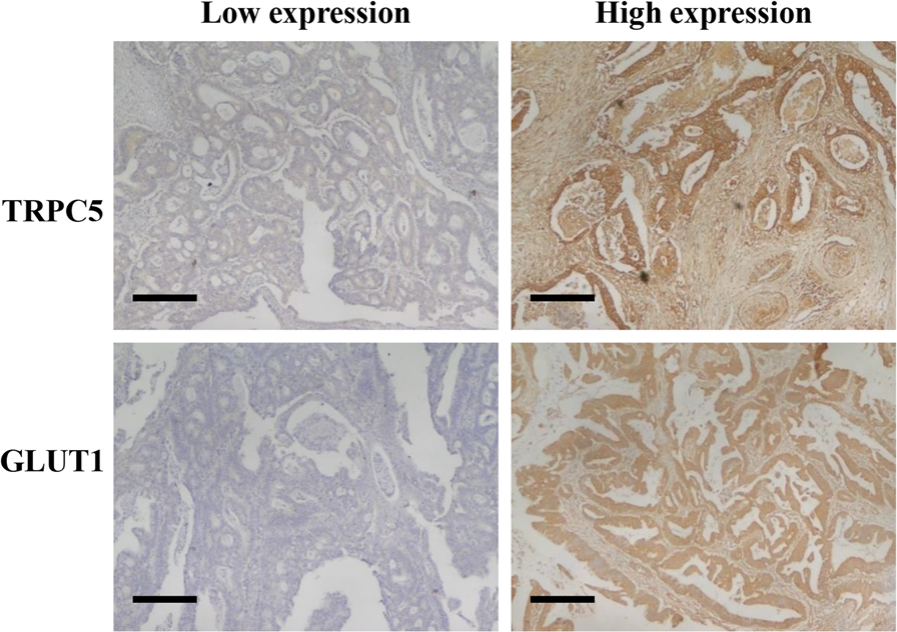
Representative images from immunohistochemical staining of TRPC5 and GLUT1 expression in human CRC tissues. Scale bars, 100 μm

**Fig. 5:**
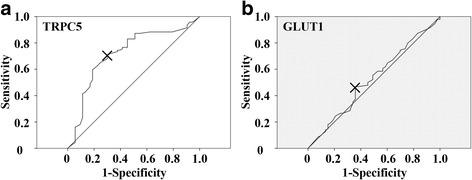
Cutoff values of TRPC5 and GLUT1 score assessed by ROC curve. The ROC curves discriminated responders from non-responders according to TRPC5 score (**a**) (AUC = 0.719; 95% CI = 0.631–0.808) with a cutoff value of 5 and GLUT1 score (**b**) (AUC = 0.524; 95% CI = 0.426–0.622) with a cutoff value of 6.3

As was shown in Table 2, among the 147 advanced CRC patients enrolled in this study, 53 patients achieved CR/PR (responders) and 94 patients achieved SD/PD (non-responders) after chemotherapy. Different levels of TRPC5 and GLUT1 protein were observed in tumor tissues from different CRC patients (Fig. 4). ROC analysis identified 5 and 6.3 as the optimal cutoff value of TRPC5 score and GLUT1 score respectively to discriminate responders from non-responders (Fig. 5). Pearson’s chi-squared test showed the positive correlation between TRPC5 and GLUT1 protein levels and a high TRPC5/GLUT1 expression was closed correlated with chemoresistance (Table 3), which was consistent with our previous findings [12]. Interesting, high TRPC5 expression was found to be significantly associated with chemoresistance only in case of high GLUT1 expression, while no association was observed between TRPC5 expression and chemotherapy outcome in the case of low GLUT1 expression (Table 4).

**Table 2:** Clinical and pathological characteristics of 147 CRC patients

Characteristic	All patients (*n* = 147)	
	*n*	%
Age (years)		
Mean	62.2	
SD	11.4	
< 65	85	57.82
≥ 65	62	42.18
Sex		
Male	63	42.86
Female	84	57.14
Tumor location		
Colon cancer	81	55.10
Rectal cancer	66	44.90
Tumor differentiation		
Well or moderately	110	74.83
Poorly	37	25.17
Outcome of chemotherapy^a^		
CR	6	4.08
PR	47	31.97
SD	59	40.14
PD	35	23.81

^a^Outcome of first-line chemotherapy in CRC patients was classified according to the Response Evaluation Criteria in Solid Tumours 1.1 (RECIST 1.1) categories (complete response (CR), partial response (PR), stable disease (SD), progressive disease (PD))

**Table 3:** Characteristics of CRC patients according to TRPC5/GLUT1 expression status

Characteristic	TRPC5			GLUT1		
	High (*n* = 83)	Low (*n* = 64)	p*	High (*n* = 64)	Low (*n* = 83)	p*
Age (years)			0.31			1
< 65	51	34		37	48	
≥ 65	32	30		27	35	
Sex			0.06			0.85
Male	30	33		28	35	
Female	53	31		36	48	
Primay tumor location			0.93			0.08
Colon cancer	46	35		30	51	
Rectal cancer	37	29		34	32	
Tumor grade			0.47			0.67
Well or moderately	64	46		49	61	
Poorly	19	18		15	22	
Chemotherapy outcome			< 0.01			0.01
responders	16	37		16	37	
non-responders	67	27		48	46	
GLUT1			< 0.01			
High	44	20				
Low	39	44				

*p < 0.05 by the chi-squared test

**Table 4:** GLUT1 expression in associated with the impact of TRPC5 expression on chemotherapy outcome in advanced CRC

		Chemotherapy outcome		
		Responders	Non-responders	p*
High GLUT1	High TRPC5	2	42	< 0.01
	Low TRPC5	14	6	
Low GLUT1	High TRPC5	14	25	0.13
	Low TRPC5	23	21	
High TRPC5	High GLUT1	2	42	< 0.01
	Low GLUT1	14	25	

*p < 0.05 by the chi-squared test

## Discussion

As the channels of Ca^2+^ influx into cell, trp channels were demonstrated to be involved in many cellular biological behaviors in cancer [23–27]. For example, TRPC1, TRPC3 and TRPC6 were proven to be participated in proliferation of multiple types of cancer, including breast caner [28, 29], ovarian cancer [30], liver cancer [31], and brain cancer [32]. Recently, up-regulation of TRPC5 expression was found to be associated with chemoresistance in human CRC [2] and breast cancer [26].

In present study, the [Ca^2+^]_*i*_ level was found to be positively associated with the TRPC5 level in chemoresistant CRC cells, which was up-regulated or decreased according to the TRPC5 expression. This indicated that TRPC5 regulates the cellular processes through alterring the Ca^2+^ influx. It has been demonstrated that [Ca^2+^]_*i*_ is an important regulator of cell apoptosis at all stages [3], and excessive elevation of calcium will trigger intrinsic apoptotic pathway [19, 20, 33]. Numerous studies showed that up-regulation of trp channels in cancer played completely different roles, varing from inducing apoptosis to enhancing survival [3]. With regard to the chemoresistance induced by the up-regulation of functional TRPC5, there should exit Ca^2+^ efflux mechanism to maintain [Ca^2+^]_*i*_ at a relatively high level not enough to trigger [Ca^2+^]_*i*_ related apoptosis.

[Ca^2+^]_*i*_ efflux is an ATP-dependent process. In nonmalignant cells, oxidative phosphorylation is the main source of ATP under physiological condition, and inhibition of mitochondrial metabolism impaired [Ca^2+^]_*i*_ homeostasis and leads to cell death [5, 6]. Aerobic glycolysis plays important roles during tumor progression, metastasis, and relapse [7, 34] through supplying ATP and metabolites [9]. Moreover, recently aerobic glycolysis derived ATP was proven to be crucial for [Ca^2+^]_*i*_ efflux and [Ca^2+^]_*i*_ homeostasis in malignant cells [4]. Thus, we intended to explore the role of glycolysis in TRPC5 induced chemoresistance in human CRC cells.

Several studies have found elevated aerobic glycolysis in chemoresistant cancer cells which was essential for maintaining chemoresistance [10, 35–37]. We also observed an increased glycolysis activity in chemoresistant CRC cells. It was generally considered that glycolytically derived ATP is crucial for chemoresistant cancer cells to cope with constant chemotherapeutic stress [10, 21], which includes enhancing drug inactivation, mutating survival-related genes, deregulating growth factor signaling pathways, increasing expression of antiapoptotic genes, and/or activating intracellular survival signaling, etc. [38]. However, the potential mechanism of glycolytically derived ATP in chemoresistance remains unclear.

In this study, inhibition of glycolysis caused a remarkable ATP production decrease, increasement of [Ca^2+^]_*i*_ level, cleaved Caspase-3 and apoptotic cells rate, and reversed the resistance to 5-Fu in chemoresistant CRC cells, while did not cause significant change in wild human CRC cells. Since [Ca^2+^]_*i*_ efflux is ATP-dependent, and elevated [Ca^2+^]_*i*_ level has been proven to trigger apoptosis [19, 20, 33], the reasonable explaination for increasement of cleaved Caspase-3 after glycolysis inhibition was the deprivation of glycolytically derived ATP and subsequent elevated [Ca^2+^]_*i*_ level. In addition, the increased cleaved Caspase-3 and apoptotic cells rate induced by 2DG could be reduced by BAPTA-AM administration. This indicated the essential involvement of increased glycolysis in TRPC5 induced chemoresistance is [Ca^2+^]_*i*_ homeostasis maintenance through supporting ATP. Further study on advanced CRC patients who received chemotherapy showed the impact of high TRPC5 expression on chemoresistance was high GLUT1 expression dependent.

In our previously study [2, 12], up-regulated expression of TRPC5 was proven to activate glycolysis through Wnt/β-catenin signaling pathway in human CRC cells. Thus, we hypothesize that TRPC5 activates Wnt/β-catenin to induce chemoresistance through mediating Ca^2+^ influx, and promoting glycolysis to provide ATP to prevent [Ca^2+^]_*i*_ overload. Thus, rather than high TRPC5, high “TRPC5-glycolysis” was more closed to chemoresistance.

## Conclusions

Aerobic glycolysis was proven to be crucial in tumorigenesis, tumor progression and metastasis [7, 34]. Here we demonstrated the role and the potential mechanism of aerobic glycolysis in chemoresistence. These findings help to understand the complicated underlying role of TRPC5 and aerobic glycolysis in chemoresistant CRC cells.

## Abbreviations

[Ca^2+^]_*i*_: Intracellular Ca^2+^
3-BP: 3-bromopyruvate
5-Fu: Fluorouracil
CR: Complete response
CRC: Colorectal cancer
DMSO: Dimethyl sulfoxide
GLUT1: Glucose transporter 1
IC50: Half maximal inhibitory concentration
MTT: 5-diphenyl tetrazolium bromide
PD: Progressive disease
PR: Partial response
RECIST 1.1: Response Evaluation Criteria in Solid Tumors 1.1
ROC: Receiver operating characteristics
SD: Stable disease
TRPC5: Transient receptor potential channel C5
